# Viral phylodynamics and the search for an ‘effective number of infections’

**DOI:** 10.1098/rstb.2010.0060

**Published:** 2010-06-27

**Authors:** Simon D. W. Frost, Erik M. Volz

**Affiliations:** 1 Department of Veterinary Medicine, University of Cambridge, Madingley Road, Cambridge, Cambridgeshire CB3 0ES, UK; 2 Department of Epidemiology, University of Michigan – Ann Arbor, 1415 Washington Heights, Ann Arbor, MI 48109-2029, USA

**Keywords:** phylodynamics, effective population size, viral evolution, coalescent, epidemiological models

## Abstract

Information on the dynamics of the effective population size over time can be obtained from the analysis of phylogenies, through the application of time-varying coalescent models. This approach has been used to study the dynamics of many different viruses, and has demonstrated a wide variety of patterns, which have been interpreted in the context of changes over time in the ‘effective number of infections’, a quantity proportional to the number of infected individuals. However, for infectious diseases, the rate of coalescence is driven primarily by new transmissions i.e. the incidence, and only indirectly by the number of infected individuals through sampling effects. Using commonly used epidemiological models, we show that the coalescence rate may indeed reflect the number of infected individuals during the initial phase of exponential growth when time is scaled by infectivity, but in general, a single change in time scale cannot be used to estimate the number of infected individuals. This has important implications when integrating phylogenetic data in the context of other epidemiological data.

## 1. Introduction

Viruses, especially RNA viruses such as human immunodeficiency virus type 1 (HIV-1), hepatitis C virus (HCV) and influenza A virus, may exhibit a great deal of genetic variation at the population level, allowing the reconstruction of viral phylogenies that reflect the past transmission of the virus. The shape of the phylogeny can tell us a great deal about population processes, such as changes in population size and geographic population structure. It can also indicate the effects of immunological processes, such as selection of escape variants (Pybus & Rambaut 2009). For example, ‘star-like’ phylogenies are typical of populations that are growing exponentially, while ‘ladder-like’ phylogenies are consistent with a model where one variant is replaced by another due to immune escape. This integration of ecological, epidemiological and evolutionary processes has been dubbed ‘phylodynamics’ (Grenfell *et al.* 2004).

**Figure 1.**
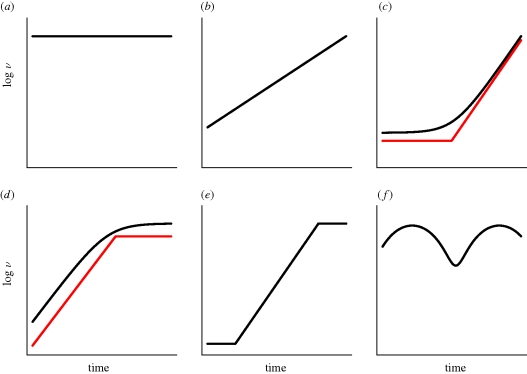
Schematic of different phylodynamic patterns for the relative size function, *ν* over time. (*a*) constant; (*b*) exponential; (*c*) piecewise expansion; (*d*) piecewise logistic; (*e*) constant–expansion–constant; (*f*) oscillatory.

Sophisticated statistical methods have been developed which allow time-stamped phylogenies to be obtained from viral sequence data (Rambaut 2000; Drummond *et al.* 2006), and these have been used in conjunction with coalescent models borrowed from population genetics (Kingman 2000; Pybus *et al.* 2000; Drummond *et al.* 2005) to determine different patterns of changes in population size over time (figure 1). These methods have been used to study the phylodynamics of many different viruses, mostly RNA viruses, but also to a lesser extent, DNA viruses (table 1). While not an exhaustive review of viral phylodynamic studies, table 1 reveals a wide range of phylodynamic patterns, ranging in complexity from a constant population size to multiple phases of growth, including oscillations. Most of these studies have used a model of the coalescent in a time-varying population, which considers the genealogical process of a small sample of taxa taken from a large population that changes in time deterministically. The population size is assumed to be homogeneous and under neutral evolution. Although in practice these assumptions are broken, it is often the case that an ‘effective population size’, *N*_e_, can be derived, which gives the same coalescence rate as an idealized population of size *N*. To date, phylodynamic studies of viral evolution have assumed that *N*_e_ is equivalent to the (effective) number of infected individuals. Although some studies argue that the effective population size may be lower than expected due to variability between individuals in infectiousness, all assume that an ‘effective number of infections’ that is proportional to the number of infected individuals.

**Table 1.** Phylodynamic patterns of viruses.

pattern	virus
constant	Canine distemper virus (Pomeroy *et al.* 2008); Hepatitis B virus (van Houdt *et al.* 2010);
	Hepatitis C virus (Golemba *et al.* 2010); HIV-1 (Deng *et al.* 2008; Tee *et al.* 2008);
	Measles virus (Pomeroy *et al.* 2008); Mumps virus (Pomeroy *et al.* 2008);
	Rabbit haemorrhagic disease virus (Kinnear & Linde 2010); Rabies virus (Hughes *et al.* 2004; Davis *et al.* 2007);
	Ross River virus (Jones *et al.* 2010); Simian foamy virus (Liu *et al.* 2008);
	St Louis encephalitis virus (Twiddy *et al.* 2003).
expansion	Hepatitis B virus (Zehender *et al.* 2008); Hepatitis C virus (Jiménez-Hernández *et al.* 2007);
	HIV-1 (Worobey *et al.* 2008); Influenza A (Goñi *et al.* 2009).
exponential	Dengue virus (Twiddy *et al.* 2003); Hepatitis C virus (Jiménez-Hernández *et al.* 2007; Pybus *et al.*^2003^, ^2005^);
	HIV-1 (Lemey *et al.* 2004; Salemi *et al.* 2005; Walker *et al.* 2005; Salemi *et al.* 2008); Influenza A (Chen & Holmes 2006; Fraser *et al.* 2009; Rambaut & Holmes 2009); Human rhinovirus (Briese *et al.* 2008);
	Measles virus (Pomeroy *et al.* 2008); Rabies virus (Hughes *et al.* 2004; Davis *et al.* 2007); West Nile virus (Snapinn *et al.* 2007).
logistic	Canine parvovirus (Pereira *et al.* 2007); Dengue virus (Carrington *et al.* 2005);
	Hepatitis B virus (Zehender *et al.* 2008); Hepatitis C virus (Verbeeck *et al.* 2006; Jiménez-Hernández *et al.* 2007).
	HIV-1 (Robbins *et al.* 2003; Hu *et al.* 2005; Walker *et al.* 2005; Bello *et al.* 2007; Tee *et al.* 2008); Human erythrovirus B19 (de Freitas *et al.* 2008);
	West Nile virus (Snapinn *et al.* 2007).
piecewise logistic	Hepatitis C virus (Tanaka *et al.* 2004; Pybus *et al.* 2003);
piecewise expansion	Hepatitis B virus (Michitaka *et al.* 2006); Hepatitis C virus (Pybus *et al.* 2005; Nakano *et al.* 2004; Kurbanov *et al.* 2007);
	Hepatitis delta virus (Kurbanov *et al.* 2007); Hepatitis E virus (Tanaka *et al.* 2006);
	HIV-1 (Kurbanov *et al.* 2003); HIV-2 (Lemey *et al.* 2003*a*); Infectious bursal disease virus (Hon *et al.* 2006);
	Japanese encephalitis virus (Twiddy *et al.* 2003); Rabies virus (Hughes *et al.* 2004)
two-phase exponential	Dengue virus (Twiddy *et al.* 2003).
nonparametric	
constant	Epizootic haemorrhagic disease virus (Biek 2007);
	St Louis encephalitis virus (Baillie *et al.* 2008).
constant/exponential phases	Avian metapneumovirus (Padhi & Poss 2009); Dengue virus (Schreiber *et al.* 2009);
	Feline immunodeficiency virus (Biek *et al.* 2006);
	Hepatitis C virus (Nakano *et al.* 2006; Njouom *et al.* 2007; Njouom *et al.* 2009; Pybus *et al.* 2009); HIV-1 (Bello *et al.* 2009; Pérez-Losada *et al.* 2010); JC virus (Kitchen *et al.* 2008);
	Rabies virus (Biek *et al.* 2007).
decline	Buggy Creek virus (Padhi *et al.* 2008); Hepatitis A virus (Moratorio *et al.* 2007);
	Hepatitis B virus (van Ballegooijen *et al.* 2009); Toscana virus (Zehender *et al.* 2009)
oscillatory	Dengue virus (Bennett *et al.* 2009); Influenza A (Rambaut *et al.* 2008); Influenza B (Chen & Holmes 2008)
	West Nile virus (Amore *et al.* in press).

Using simple epidemiological models, we have recently demonstrated that the coalescence rate of an infectious disease is related to the rate of transmission (i.e. the incidence) and not directly to the absolute number of infected individuals (i.e. the prevalence; Volz *et al.* 2009). Prevalence does affect the shape of the phylogeny, but only indirectly through sampling effects; when a higher proportion of infected individuals are sampled, more coalescent events are evident near the tips of the phylogeny. In this study, we examine whether there are conditions under which the coalescence rate may indeed reflect the ‘effective number of infections’, by comparing coalescence in epidemiological models with classical population genetics models. We also address how the conclusions of previous studies may be affected by interpreting phylodynamic patterns as being driven by incidence rather than prevalence.

## 2. Phylodynamic patterns under different epidemiological scenarios

### (a) The time-varying coalescent model

The model used most commonly for viral phylodynamics is the time-varying coalescent model (Griffiths & Tavaré 1994), which considers the genealogical process in a population that changes size in a deterministic fashion according to some relative size function, *ν*(*τ*), where *τ* is the time measured in generations, starting with the present and going backwards. For example, for a constant population size, we have *ν*(*τ*) = 1. A variety of different parametric models have been proposed for *ν*(*τ*), including a constant population size, exponential growth, logistic growth and expansion growth. These can be strung together in series to make more complicated patterns. In addition, a number of ‘nonparametric’ models have been proposed for *ν*(*τ*) (Pybus *et al.* 2000; Strimmer & Pybus 2001; Drummond *et al.* 2005; Opgen-Rhein *et al.* 2005; Minin *et al.* 2008), which when fitted to data have sometimes demonstrated complex patterns, including oscillatory dynamics (table 1).

So, what does the relative size actually mean, and how does it relate to the coalescence rate? Let us consider a sample of *n* individuals taken at time *τ* = 0, and assume that the sample can be traced back to a single common ancestor with probability 1 (i.e. ∑_0_^∞^ *ν*^−1^(*τ*)d*τ* = ∞). The dynamics of the number of distinct ancestors of the sample at time *τ* is modelled as a stochastic process {A_*n*_(*τ*), *τ* ≥ 0}, which starts at *A*_*n*_(0) = *n*, and moves down in steps of 1 until reaching 1, at which point the sample has been traced back to a most recent common ancestor. In a small time step *h*, the transition probabilities are determined by the following:
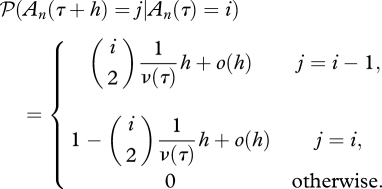



Equation (2.1) shows that the rate of coalescence increases with the number of distinct ancestors, and decreases with a greater relative size. Under a Wright–Fisher model of a haploid population, the relative size function is simply the population size, i.e. *ν*(*τ*) = *N*(*τ*). This is only an approximate result for the Wright–Fisher model, however, which holds when the sample size is small relative to the population size, as equation (2.1) assumes that only one coalescence can occur at a time. When a large proportion of the population is sampled, multiple coalescent events may occur in a single generation. In such a case, more general coalescent models that the commonly used Kingman coalescent may be more appropriate, which allow multiple ‘collisions’ of lineages (Pitman 1999; Sagitov 1999, 2003; Schweinsberg 2000; Mohle & Sagitov 2001). Although populations may deviate from the assumptions of a Wright–Fisher model—for example, they may show geographical structure—in many, but not all cases, the relative size function can be assumed to be proportional to the population size, in which case, it is referred to as the ‘effective population size’, *N*_e_, and the relative size function is *ν*(*τ*) = *N*_e_(*τ*).

If *g*_*i*_ is the length of time during which the ancestral process is in state *A*_*n*_ = *i* and *τ*_*i*_ is the time that the interval starts, then under model (2.1), *g*_*i*_ is distributed as follows (Pybus *et al.* 2000).2.1
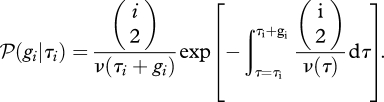



Since *P*(*g*_*i*_|*τ*_*i*_) depends only on the relative size function, equation (2.1) allows coalescent intervals to be simulated for a given relative population density *ν*(*τ*), and also allows the model to be fitted to coalescent intervals estimated from phylogenetic trees. Although branch lengths in a phylogeny are typically in units of expected substitutions per site, in many viral phylodynamic studies, a strict or ‘relaxed’ molecular clock is often used in conjunction with serial samples of sequences (Rambaut 2000; Seo *et al.* 2002*a*,*b*; Lemey *et al.* 2003*b*; Sanderson 2003; Drummond *et al.* 2006; Yang *et al.* 2007), such that branch lengths are scaled in absolute time. Many studies do not assume a specific generation time, and in doing so, generate estimates of the product of the generation time and *ν*(*τ*) as the ‘effective population size’. To avoid making assumptions regarding how time is rescaled, some studies simply refer to estimates of *ν*(*τ*) obtained from the data as ‘genetic diversity’ (Carrington *et al.* 2005; Rambaut *et al.* 2008; Schreiber *et al.* 2009; van Ballegooijen *et al.* 2009).

### (b) Deterministic models for the coalescent

A common framework for modelling infectious diseases is compartmental models, in which the population is divided up into subpopulations called *compartments*, such as susceptible and infected individuals. The rate of change in the size of these compartments as we go forward in time, *t*, is modelled using differential equations. We can also consider a differential equation for the dynamics of the number of lineages over time based on equation (2.1).2.2
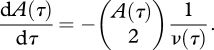



There are two different ways of interpreting this equation. Firstly, we could consider *A* as an approximation to the number of lineages when the sample size is very large i.e. *A* = lim_*n*→∞_*A*_*n*_, in which case we could approximate equation (2.2) by −*A*(*τ*)^2^/ 2*ν*(*τ*), as *n*(*n* − 1) tends to *n*^2^ as *n* gets large. This approximation is surprisingly good, even when the number of distinct lineages is small (e.g. only an 11% difference when *n* = 10). Another way to look at *A* is as an approximation to the mean number of lineages over time i.e. *A* ≈ *E*(*A*_*n*_); we adopt the latter interpretation. Recently, we showed that for many simple epidemiological models, the rate of coalescence in a phylogeny is a function of the number of infected individuals, *Y* and the rate at which susceptible individuals, *X*, become infected, *f*_*XY*_. If we denote time going backwards from the present as *s*, the dynamics of the number of ancestral lineages over time can be modelled using the following differential equation (Volz *et al.* 2009).2.3
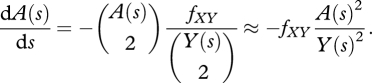



The rationale underlying equation (2.3) is that coalescence occurs at a rate equal to the transmission rate, *f*_*XY*_; coalescence can occur between any pair of infected individuals, but will only result in a decrease in the number of lineages in the sample if both the source of infection and the recipient of infection are sampled, either directly (through these individuals being included in the sample) or indirectly (through sampling their descendant viral lineages). In our previous work, we modelled the number of lineages using expression (2.3); in order to assist comparisons between the coalescent and epidemiological models, we assume that the population size is large, such that 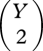 ≈ *Y*^2^/2, but not the number of samples. Hence, we model the number of lineages over time as follows.2.4
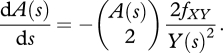



Note that the time scale in equation (2.4) is in real time, and the coalescence rate is determined by a combination of the number of new infections per unit time (the absolute incidence) and the level of sampling (which, for a fixed sample size, is dependent on the absolute prevalence of infection). The term 2*f*_*XY*_/*Y*(*s*)^2^ on the right-hand side of equation (2.4) is simply the probability that a pair of ancestral lineages are descended from a common ancestor, and this probability is the same as that under a Moran model, because one of the lineages we are following must be the ‘offspring’ and the other must be the ‘parent’, and there are two ways for this to occur. This is in contrast to the haploid Wright–Fisher model, in which the probability of a pair of ancestral lineages being descended from a common ancestor is the inverse of the population size. Despite being based on differential equations, extensive simulation results show that this model is surprisingly good at recapitulating the dynamics of the number of lineages over time (at least on average) for a range of population sizes and sample sizes (Volz *et al.* 2009 and this study), although it should be noted that the variance in the number of lineages can be large. Although this may be an issue when trying to estimate parameters from data (for example, using equation (2.1)), equation (2.4) is extremely useful to help understand the connection between the epidemiological and evolutionary dynamics. To illustrate this, we considered the dynamics of the number of lineages over time for a variety of epidemiological scenarios using two simple, but commonly used, epidemiological models.

### (c) A model with a constant number of infected individuals

A useful ‘null model’ to study the change in effective population size over time is a model with a constant population size. In an epidemiological model, this corresponds to an endemic equilibrium. As an example of a model with an endemic equilibrium, we consider a simple model commonly used to study the spread of HIV among men who have sex with men (for a comparison of the deterministic and stochastic version of this model, see Jacquez & Simon 1993). If *X* denotes the number of susceptible individuals and *Y* denotes the number of infected individuals, the rates of change of *X* and *Y* are as follows:2.5
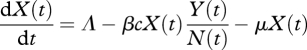

and2.6
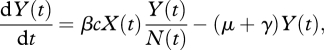

where2.6
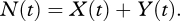



**Figure 2.**
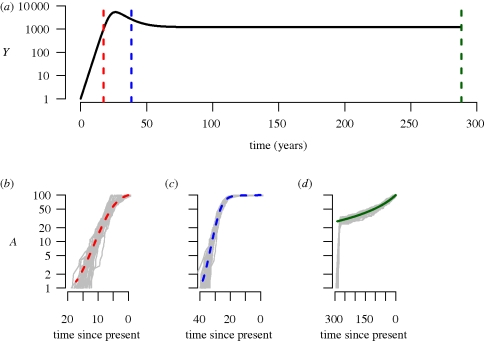
Phylodynamics of a simple susceptible-infected model in an open population (equations (2.7) and (2.8) in the main text). (*a*) Dynamics of the number of infected individuals, *I* over time in years. The vertical lines denote sampling times, and the number of lineages over time (*b*) during exponential growth (red), (*c*) following the peak of infected individuals (blue) and (*d*) at equilibrium (green). The grey lines represent stochastic simulations; in order to generate a fair comparison between the deterministic model and the stochastic simulations, time was shifted for each simulation such that the peak prevalence occurred at the same time as in the deterministic model. Parameter values are as follows (with time in years); *βc* = 52, *γ* = 1/10, *μ* = 1/70, **Λ** = 10000/70. The initial conditions were: *X*(0) = 9999, *Y*(0) = 1. Sampling times were set at 900/52, 2000/52 and 15000/52 years, and a sample size of 100 was assumed, i.e. *A* = 100. Numerical simulations were performed in R (R Development Core Team 2009) using the simecol library (Petzoldt & Rinke 2007). Stochastic simulations were performed with SimPy (http://simpy.sourceforge.net). All code is available from S.D.W.F. on request.

Here, *β* denotes the probability of infection per contact, *c*, the contact rate, *μ*, the natural mortality rate, *γ*, the excess mortality caused by infection, and **Λ**, the rate of immigration/birth of new susceptibles. The dynamical behaviour of the model depends on the value basic reproductive number *R*_0_ = *βc*/*μ* + *γ*. If *R*_0_ > 1, the number of infected individuals initially increases exponentially, plateaus, and finally reaches an equilibrium (figure 2).

By substituting *f*_*XY*_ = *βcXY*/*N* into equation (2.4), we obtain the following expression.2.7
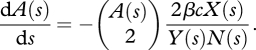



If we denote the equilibrium population sizes of the number of susceptibles, infecteds and the total population size as X*, Y* and N* = X* + Y*, respectively, the rate of change of lineages going backwards in time, d*A*/d*s*, is as follows.2.8
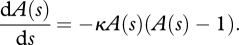

The solution of which is2.9
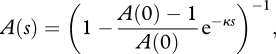

where2.10
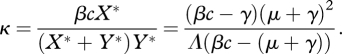



Equation (2.8) shows that the coalescence rate is not proportional to the number of infected individuals, but is also a function of the number of susceptible individuals. Consequently, even for this relatively simple model, the expression (2.10) for the rate parameter *κ* is a nonlinear combination of several parameters, and shows that in the absence of other information about the epidemiological processes, the dynamics of lineages through time may provide very little information about individual parameters. Note that by starting the system at equilibrium, the number of infected individuals going backwards in time is constant i.e. all information on when the susceptible population was invaded with an infected individual is lost.

We compared the number of lineages over time using equation (2.9) with stochastic simulations (figure 2). The analytical solution gives a good approximation to the mean number of lineages over time for the period during which the system is close to equilibrium.

### (d) An exponentially growing population

During the early phase of epidemic growth, when *X*(*t*)/*N*(*t*) ≈ 1, the number of infected individuals increases exponentially over time.2.10
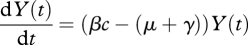

and
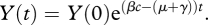

Going backwards in time, the expressions for *Y*(*s*) and *A*(*s*) are as follows:
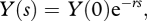


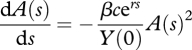

and
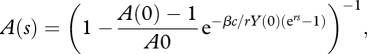

where
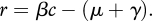



It is also informative to examine the expression for d*A*(*s*)/d*s* as a function of *Y*(*s*) in the case of exponential growth.2.11
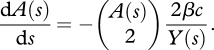



During exponential growth, there is a linear relationship between the prevalence and the incidence, and hence the coalescence rate is directly proportional to the number of infected individuals.

### (e) Logistic growth

The model given by equations (2.5) and (2.6) also exhibits similar dynamics to logistic growth. Although closed expressions for *X*(*t*) and *Y*(*t*) cannot be obtained for this model, we can obtain the number of lineages through time by numerically solving for *A* backwards in time, either by simulating the complete system of differential equations backwards (as in Volz *et al.* 2009), or by simulating the epidemic forwards in time, and storing *f*_XY_ and *Y*, which can then be used as inputs into a single differential equation for *A*. Figure 2 demonstrates that the number of lineages over time, for a sample taken just after peak prevalence, is well described on average by the differential equation model (2.5) and (2.6). During exponential growth, incidence is high and lineages increase rapidly, while after the peak, incidence is low, and the rate of increase of lineages drops.

### (f) Relationship between coalescence rate and estimates of effective population size

**Figure 3.**
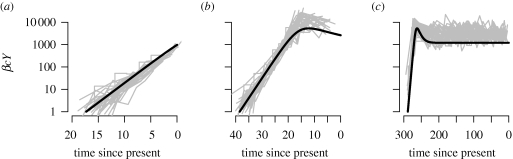
The product of the transmission probability, contact rate and number of infected individuals, *βcY*, at different stages of the epidemic depicted in figure 2 (smooth black line) obtained from numerically solving equations (2.7) and (2.8), along with numerical estimates of ‘effective population size’ estimated using generalized skyline plots fitted to stochastic simulations (grey lines) on the same scale. During the exponential growth period, the skyline generates good estimates of *βcY*. Parameter values and initial conditions are as described in figure 2. Skyline plots were generated using the APE library (Paradis *et al.* 2004) in R (R Development Core Team 2009). (*a*) Exponential growth; (*b*) after peak; (*c*) at equilibrium.

Many previous studies have estimated the ‘effective population size’, *N*_e_ of an epidemic without considering an explicit model of disease transmission. To investigate the relationship between estimates of effective population size obtained using standard coalescent models, transmission rates, and number of infected individuals, we fitted generalized skyline plots to stochastic simulations of the model based on equations (2.5) and (2.6). When branch lengths in the phylogeny are measured in continuous time, as is common for viral phylodynamic studies, then assuming model (2.1), the use of this approach will generate estimates of the product of the generation time and *N*_e_. From a comparison of equations (2.2) and (2.11), it might initially appear that the application of standard coalescent models would give estimates of 2*βcY*. However, as shown in figure 3, during exponential growth, the skyline is a good estimate of *βcY*. This arises as epidemiological models that operate in continuous time bear a closer resemblance to the Moran population model, where generations overlap in continuous time and only one coalescent event can occur at a time. The ‘coalescent effective population size’, defined as the average time to a coalescent event measured in units of the average time back to a birth event is *N*_e_ = *N* for a Wright–Fisher model, and *N*_e_ = *N*/2 for a Moran model (Wakeley & Sargsyan 2009). Consequently, we have to halve the estimates of effective population size obtained assuming a Wright–Fisher model. In addition, the appropriate scaling in time is determined by the infectivity, which determines the average time back to a transmission event (analogous to a birth event), and not by the duration of infectiousness. Figure 3 demonstrates that standard models perform well in terms of both the absolute number of infected individuals, and the rate of change over time, suggesting that previous studies may have obtained good estimates of epidemic doubling time, despite making the erroneous assumption that coalescence is directly related to prevalence. However, as the relationship between the transmission rate, *f*_*XY*_ and the number of infected individuals *Y* is different during exponential growth and at equilibrium, we cannot find a single transformation of time such that the coalescence rate corresponds to the number of infected individuals over the entire epidemic. In this model, as the time between infections changes, the use of a single transformation of time to fit the early stages of the epidemic results in an overestimation of the true number of infected individuals in the later stages.

### (g) Oscillatory dynamics

By application of the non-parametric ‘skyline’ type approaches used in the previous section, a number of studies have demonstrated oscillations in the relative size, *ν*, over time. Oscillations in the number of infected individuals in an epidemiological model can arise, for example, from seasonally changing contact rates. A simple example of this, appropriate to study the dynamics of an acute infection under seasonality, which considers susceptible, *X*, infected, *Y*, and immune individuals, *Z*, is as follows.2.12
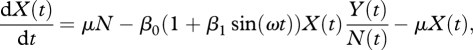

 2.13
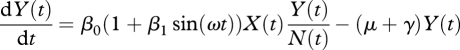

and2.14
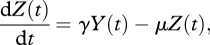

where
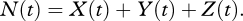



**Figure 4.**
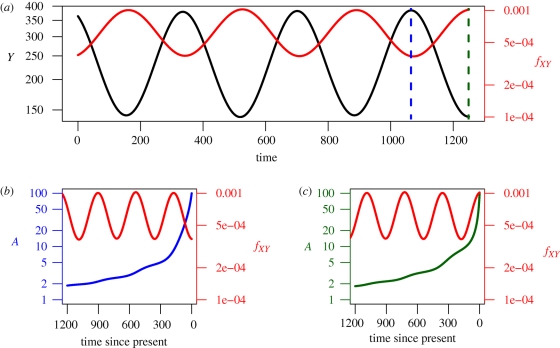
(*a*) Dynamics of the number of infected individuals, *Y*, and the transmission rate *f*_*XY*_ for a susceptible-infected-recovered model with seasonal forcing, given by equations (2.20)–(2.22) in the main text. Parameter values are as follows (with time in days): *β*_0_ = 10/7, *β*_1_ = 0.05, *ω* = 2*π*/365, *γ* = 1/7, *μ* = 1/25550. The population size, *N*, was assumed to be 10^6^. Initial conditions: *S* = 100029.946, *I* = 142.978, *R* = 899827.076. (*b*) The time of sampling for the high prevalence scenario was *t* = 3465, when *I* = 384.477 (38.4 per 100 000) (*c*) and the time of sampling for the low prevalence scenario was *t* = 3649, when *I* = 140.7068 (14.0 per 100000).

We chose parameter values that gave annual fluctuations in the number of infected individuals, and numerically simulated the epidemic over a ten year period. We then simulated the dynamics of the number of lineages, sampling at the last peak of infection. Figure 4 shows the prevalence of infection, *Y*(*t*) over time. This looks very different from the transmission rate, *f*_*XY*_ = *β*_0_ (1 + *β*_1_ sin(*ωt*))*X*(*t*)*Y*(*t*)/*N*(*t*), which determines the rate at which lineages coalesce. If we were to mistakenly interpret the coalescence rate as proportional to the number of infected individuals, we would conclude that the prevalence was at a peak when it was in a trough, and vice versa, as for these parameter values, *Y*(*t*) and *f*_*XY*_ are out of phase. For more complex oscillatory dynamics, such as biennial cycles, the relative magnitudes of *Y*(*t*) and *f*_*XY*_ may also differ. These model results also reinforce previous assertions (Rambaut *et al.* 2008; Stack *et al.* in press), that a sample taken at a single point in time may provide relatively little information about the past population dynamics, as the population bottlenecks result in all sequences sampled at a single timepoint having a relatively recent common ancestor.

## 3. Discussion

Using simple differential equation based models to gain insights into the phylodynamics of viral infections, we have demonstrated that the pattern of coalescence for an infectious disease is dominated by the transmission rate, while the number of infected individuals is of secondary importance. Although Holmes *et al.* (1995) recognized that coalescence in an infectious disease was related to transmission, this was not taken into account in later phylodynamic studies, which referred to the ‘effective number of infections’, i.e. the prevalence. Some studies also noted that the generation time is effectively the time between infections (Pomeroy *et al.* 2008; van Ballegooijen *et al.* 2009), and not the duration of infectiousness, but did not recognize that this changes throughout an epidemic. Hence, a single transformation of time, which is commonly used to estimate *N*_e_ from temporally sampled sequence data, cannot be used to recover the ‘effective number of infected individuals’. In some cases, such as during exponential growth, there is a linear relationship between the transmission rate and the number of infected individuals, and with an appropriate choice of time scale (dividing time by *βc* in the models here) it is possible to estimate the number of infected individuals, but this is not true in general. Some studies (e.g. Rambaut *et al.* 2008) have been vague in the interpretation of the coalescence rate, relating it to ‘genetic diversity’. We believe that this is a little too cautious—the rate of coalescence can be related to epidemiological parameters, but we have to explicitly consider the underlying transmission dynamics for this to be done correctly. For example, in the case of endogenous retroviruses (Romano *et al.* 2008), the transmission tracks the reproduction of the host, and standard coalescent models used for human populations can be used. In the case of viruses where there is significant vertical and horizontal transmission, more sophisticated models that incorporate coalescence in both the host and the virus will be required to interpret the phylodynamics patterns in the context of transmission parameters. A particularly pertinent quote comes from a review by Donnelly & Tavaré (1995) in their discussion of the time-varying coalescent (equation (2.1)):

[T]he results described above do not apply in general. It is true for very general neutral models that unless there are discontinuities, i.e. sudden changes, in the processes governing the population size, the ancestral process can be represented as a time change of the process described in (equation (2.1)). However, the form of the time change, which is in general different from (equation (2.1)), depends on properties of the random process governing the rate at which individuals are born in the population, about which little is known in many practical contexts. It thus appears that some caution is appropriate in applying the above results on the coalescent in populations of variable size.

(Donnelly & Tavaré 1995, p. 408)

That coalescence is related to transmission has important implications when interpreting phylodynamic patterns in the context of other data, such as information on the timing of external events or on disease prevalence. For example, in a recent study of dengue (DENV-4) in Puerto Rico (Bennett *et al.* 2009), although both *N*_e_ and case counts fluctuated over time, changes in *N*_e_ preceded changes in case counts by about seven months. This puzzling result is easily explained when one recognizes that the coalescence rate is a measure of incidence; as shown in our simple model of an oscillating epidemic, we expect incidence and prevalence to be out of phase, and in general, peaks of incidence precede peaks of prevalence. There was also no simple relationship between the amplitude of the fluctuations in *N*_e_ compared with the amplitude in case counts; in order to derive a meaningful comparison between these data, we would have to compare fluctuations in estimated incidence with *N*_e_. Multiple studies have interpreted the timing of changes in phylodynamic patterns in the context of changes in other factors. For example, a decline in a skyline plot obtained from hepatitis A sequences sampled in France coincided with the introduction of vaccination (Moratorio *et al.* 2007), while a massive expansion in the ‘effective number of infections’ of hepatitis C virus in Egypt fell within a time period when the general population was treated with parenteral antischistosomal treatment (Pybus *et al.* 2003). Such external forces have a more immediate impact on transmission than prevalence.

The phylodynamic patterns can also be affected by sampling; sampling a higher fraction of the infected individuals at a time results in more recent coalescent times, and shorter terminal (external) branches of the tree, and a different tree shape (Mooers 1995; Rannala *et al.* 1998; Pybus *et al.* 2000, 2002; Purvis & Agapow 2002; Huelsenbeck & Lander 2003; Volz *et al.* 2009). As many viral phylodynamic studies employ serial samples of viral sequences, it is important to correct for possible differences in sampling depth, which will be a function ofthe temporal pattern of the sampling and the number of infected individuals. In a heterogeneous epidemic, the extent to which specific subpopulations are over- or under-sampled also has to be taken into account. The model framework we present here can be extremely informative to help understand the potential effects of sampling on phylodynamic patterns, and offers a more computationally faster approach to studying sampling effects than approaches based on full epidemic simulations coupled with computationally intensive Bayesian approaches for estimating *N*_e_ (Stack *et al.* in press).

Deterministic models of the phylodynamics of infectious disease can be very informative due to their relative simplicity. However, in some cases, such as the very early stages of an epidemic, or an endemic infection in a small population, a stochastic model may be more appropriate. In the simple case of a susceptible-infected (SI) model in a closed population (i.e. equations (2.5) and (2.6) with **Λ** = *μ*= 0), the timing of the coalescent events coincides with each transmission, and hence in this case, we can use the widely studied stochastic version of the SI model to model changes in ancestral lineages through time. However, in general, we cannot simply borrow from the epidemiological or population genetic literature. Most work on coalescent theory in finite populations has focused on birth–death processes (Hey 1992; Nee *et al.* 1994; Rannala 1997), either homogenous or non-homogenous, which are too simple for our purposes, while stochastic epidemiological models generally consider the dynamics of the process forward in time, rather than backwards, and do not consider the number of lineages. Unlike the deterministic models, in general we cannot simply run the nonlinear epidemiological models backwards in time from the present; for example, the stochastic version of the model (2.5) and (2.6) reaches a quasistationary state, at which point, the system has no ‘memory’ of when the first infection occurred.

The simple nature of the epidemiological models considered here allowed us to draw direct comparisons between population genetics models such as the Wright–Fisher and the Moran model, and epidemiological models. The correspondence between population genetic and epidemiological models becomes more complex in the case of heterogeneous populations; the models described here can be extended to consider heterogeneous populations, such as different contact rates, different infectivities, spatial structure and so on. For example, previously we considered a model of HIV infection which assumed two stages of infection, a brief, highly infectious acute period, followed by a long, less infectious chronic period (Volz *et al.* 2009), such that there is no longer a single rate of coalescence that applies to all individuals. In addition, for the simple models discussed here, the shape of the tree is captured by the dynamics of the number of lineages over time. However, phylogenetic trees contain more information than simply the number of lineages over time, for example tree balance, the distribution of the length of the terminal branches, and in the case of a heterogeneous population, the relative distribution of subpopulations across the tree. The development of new phylodynamic models will help to elucidate the role of epidemiological processes in generating these patterns.
